# Prevalence of Loneliness and Its Association With General and Health-Related Measures of Subjective Well-Being in a Longitudinal Bicultural Cohort of Older Adults in Advanced Age Living in New Zealand: LiLACS NZ

**DOI:** 10.1093/geronb/gbac087

**Published:** 2022-06-29

**Authors:** Roy Lay-Yee, Barry J Milne, Valerie A Wright-St Clair, Joanna Broad, Tim Wilkinson, Martin Connolly, Ruth Teh, Karen Hayman, Marama Muru-Lanning, Ngaire Kerse

**Affiliations:** Centre of Methods and Policy Application in the Social Sciences (COMPASS), School of Social Sciences, Faculty of Arts, University of Auckland, Auckland, New Zealand; Centre of Methods and Policy Application in the Social Sciences (COMPASS), School of Social Sciences, Faculty of Arts, University of Auckland, Auckland, New Zealand; Department of Statistics, Faculty of Science, University of Auckland, Auckland, New Zealand; AUT Centre for Active Ageing, Auckland University of Technology, Auckland, New Zealand; Freemasons Department of Geriatric Medicine, School of Medicine, University of Auckland, Auckland, New Zealand; Department of Medicine, University of Otago, Christchurch, New Zealand; Department of Medicine, School of Medicine, Faculty of Medical and Health Sciences, University of Auckland, Auckland, New Zealand; Department of General Practice & Primary Health Care, School of Population Health, Faculty of Medical and Health Sciences, University of Auckland, Auckland, New Zealand; Department of General Practice & Primary Health Care, School of Population Health, Faculty of Medical and Health Sciences, University of Auckland, Auckland, New Zealand; James Henare Māori Research Centre, University of Auckland, Auckland, New Zealand; Department of General Practice & Primary Health Care, School of Population Health, Faculty of Medical and Health Sciences, University of Auckland, Auckland, New Zealand

**Keywords:** Health-related quality of life, Loneliness, Self-rated health, Social support

## Abstract

**Objectives:**

There is evidence that loneliness is detrimental to the subjective well-being of older adults. However, little is known on this topic for the cohort of those in advanced age (80 years or older), which today is the fastest-growing age group in the New Zealand population. We examined the relationships between loneliness and selected subjective well-being outcomes over 5 years.

**Methods:**

We used a regional, bicultural sample of those in advanced age from 2010 to 2015 (Life and Living in Advanced Age: a Cohort Study in New Zealand). The first wave enrolled 937 people (92% of whom were living in the community): 421 Māori (Indigenous New Zealanders aged 80–90 years) and 516 non-Māori aged 85 years. We applied standard regression techniques to baseline data and mixed-effects models to longitudinal data, while adjusting for sociodemographic factors.

**Results:**

For both Māori and non-Māori, strong negative associations between loneliness and subjective well-being were found at baseline. In longitudinal analyses, we found that loneliness was negatively associated with life satisfaction as well as with mental health-related quality of life.

**Discussion:**

Our findings of adverse impacts on subjective well-being corroborate other evidence, highlighting loneliness as a prime candidate for intervention—appropriate to cultural context—to improve well-being for adults in advanced age.

Humans are social beings who need connection with one another to survive and thrive (Cacioppo et al., 2008). In these times of global pandemic, social connection and cohesion are threatened on a massive scale (Kivi et al., 2021; van Tilburg et al., 2021). As well as their health, the well-being of people and the communities in which they live are in jeopardy. Given this context, loneliness has come to the forefront of public awareness, particularly in the western world, manifesting an extreme form of disconnection experienced as a negative emotional state (Morgan et al., 2020a). In conceptual terms, an individual’s loneliness can be defined as arising from a perceived gap between the desired and actual state of social relationships (Perlman & Peplau, 1981). Loneliness differs from social isolation because it is not only the contact but its quality that are definitive. In a sense, loneliness is a normal part of living: it can affect individuals at any age (Hawkley & Cacioppo, 2007), though the specific experience may vary with the needs and circumstances of each life stage (Rokach, 2000). Older adults may be particularly prone to loneliness given they are more likely to be retired; to suffer personal loss of a spouse, family, and friends; and to experience reduced functional status (Cohen-Mansfield et al., 2016); while social exclusion may also be at play (Scharf et al., 2005). Those who become lonely bear the harmful consequences of greater morbidity and mortality risk (Courtin & Knapp, 2017; Holt-Lunstad et al., 2015). The subjective—rather than objective—well-being of older adults is arguably a more salient outcome to examine. From the perspective of older adults themselves, having good social relationships and support, engaging in social activities, and retaining a social role are key elements to good quality of life (Gabriel & Bowling, 2004). From a societal point of view, older adults continue to contribute through employment, volunteering, mentoring, and supporting family members. Thus, for the benefit of older adults, and the substantial social dividend they provide, it is important that their well-being is protected and fostered; to do that, we must understand the underpinnings of their well-being. We propose to address the relationship between loneliness and indicators of subjective well-being in older adults aged 80 years or older.

Current research on this topic has focused on older adults aged 65+ years in largely cross-sectional studies. Loneliness has been found to be negatively associated with general quality of life in studies of community-dwellers (Gerino et al., 2017; Szabo et al., 2019); the visually impaired (La Grow et al., 2015; Renaud et al., 2010), and chronically ill rural-dwellers (Theeke & Mallow, 2013); and with life satisfaction in studies of community-dwellers (Beller & Wagner, 2018), primary care attendees (Golden et al., 2009), and university program attendees (Tomas et al., 2019). Loneliness has also been shown to be detrimental to self-rated health (SRH) of community-dwellers (Holtfreter et al., 2016; La Grow et al., 2012; Nummela et al., 2011); and to health-related quality of life (HRQOL) of community-dwellers (Freak-Poli et al., 2022; Tan et al., 2020), caregivers (Ekwall et al., 2005), the sicker (Musich et al., 2015), the visually impaired (Renaud et al., 2010), and low-income earners (Wippold et al., 2021).

Despite growing evidence on older adults in general, a gap exists with respect to loneliness among those of advanced age and, in particular, among older Indigenous populations. Indigenous people aged 80 years or older form the fastest-growing age group in western societies. In New Zealand, for example, the 65+ age group increased from 9.9% to 14.3% of the total population from 1981 to 2013, projected to rise to 23.8% by 2043; meanwhile, the 85+ subgroup comprises an increasing proportion of the 65+ group, from 7.5% in 1981 to 12.1% in 2013, projected to rise to 19.7% by 2043 (Statistics New Zealand, 2015). The older (65+) Māori (Indigenous) population of New Zealand is also growing with its proportion of the total population rising from 3.3% in 1991 to 9.4% in 2018 (Statistics New Zealand, 2018). A national social survey conducted in 2012–2013 found that among Māori adults, 32.6% were lonely compared to 29.6% of European adults (Statistics New Zealand, 2013). While people of advanced age represent a growing section of society, their societal impacts will become greater in terms of not only public expenditure but also their treasured contributions to families and communities; thus, enhancing the well-being of those in advanced age will benefit society as well as the individuals themselves.

Addressing the New Zealand case, this paper aims to investigate the association between loneliness and subjective well-being, while considering sociodemographic factors. The paper’s unique contribution is in exploring these relationships in a bicultural cohort of Māori and non-Māori of advanced age followed over time from 2010 to 2015 (Kerse et al., 2015). Māori are the Indigenous people of New Zealand—with special status enshrined in the Treaty of Waitangi (Te Tiriti o Waitangi), the founding document of the nation—who have historically borne, and continue to bear, the burden of social and health inequities (Dyall et al., 2014). Knowing more about the adverse outcomes associated with loneliness and their sociocultural context adds substance to the urgency for and design of appropriate interventions and their evaluation (Lim et al., 2020). Further, longitudinal investigation is important to shed light on the dynamics of loneliness and its effects (Mund et al., 2019).

*Research question 1*. What is the baseline prevalence of loneliness among older adults of advanced age by ethnic group, and does this change over time?
*Research question 2*. At baseline, is loneliness associated with general and health-related well-being (adjusted for sociodemographic factors) by ethnic group? We hypothesize that loneliness is negatively associated with general and health-related well-being outcomes.
*Research question 3*. Do any associations found at baseline, adjusted for sociodemographic factors, persist longitudinally? We hypothesize that any baseline associations will not change over time.

## Method

### Data Source

The data for analysis were taken from LiLACS NZ (“Life and Living in Advanced Age: a Cohort Study in New Zealand” or “Te Puāwaitanga o Ngā Tapuwae Kia Ora Tonu” as described in the Māori language). LiLACS NZ is a bicultural cohort followed annually from 2010 to 2015 (Kerse et al., 2015). Six waves of data were collected by interview using a questionnaire assessing health and well-being. All Māori aged 80–90 years and all non-Māori aged 85 years living in one geographical region of New Zealand (Bay of Plenty and Lakes District Health Board areas, excluding Taupo) were invited to participate in the study. To enable sufficiently powered ethnic-specific analyses, approximately equal numbers were recruited of non-Māori aged 85 years and Māori aged 80–90 years (eligible age was extended as Māori have lower life expectancy and lower population proportion). The baseline (Wave 1, 2010) enrolled 937 people (92% of whom were living in the community): 421 Māori and 516 non-Māori. Participants undertook either a full questionnaire that included an item on loneliness (*n* = 671: 267 Māori and 404 non-Māori) or answered some core questions only (*n* = 261: 150 Māori and 111 non-Māori). This paper analyzes the data on participants with information on loneliness. LiLACS NZ was approved by the Northern X Regional Ethics Committee (approval number: NTX 09/09/088) and all study participants provided written informed consent.

### Description of Variables

#### Loneliness

There was a single question on loneliness: “Would you say that you—always, often, sometimes, or never feel lonely?” Participants were asked to indicate one of the following valid response categories: never, sometimes, often, or always. We found that 5.1% of Māori and 5.5% of non-Māori reported “always” or “often” feeling lonely. To increase numbers for analysis, we adopted a binary working definition of loneliness, that is, “lonely” (always, often, or sometimes) and “not lonely” (never).

#### Sociodemographics (at baseline)

- Age group: <85, ≥85; note all non-Māori were aged 85 years at the start of the study.
- Gender: men, women.
- Highest educational qualification: none/primary, any secondary, postsecondary.
- Main lifetime occupation of the participant or their spouse: professional, technical/trade, other.
- Marital status: partnered, widowed, separated/divorced/never partnered. (Longitudinal data available.)
- Retired from paid work: no, yes.

#### Outcomes (at baseline)

Data for outcomes were available at baseline (2010). Where longitudinal data (2010–2015) were also available, this is indicated in the variable descriptions below.

#### General well-being

Two questions assessed levels of life satisfaction and quality of life, respectively, on a 5-point scale.

- Life satisfaction: All things considered, how satisfied are you with your life as a whole these days?
  1 = very dissatisfied, 2 = dissatisfied, 3 = neither satisfied nor dissatisfied, 4 = satisfied, and 5 = very satisfied. (Longitudinal data available.)
  Responses were heavily skewed toward higher ratings. For analysis purposes, we created a binary variable: “very satisfied” (Category 5) versus “not very satisfied” (combining Categories 1–4).
- Quality of life: How would you rate your quality of life?
  1 = very poor, 2 = poor, 3 = neither good nor poor, 4 = good, and 5 = very good.
  Responses were heavily skewed toward higher ratings. For analysis purposes, we created a binary variable: “very good” (Category 5) versus “not very good” (combining Categories 1–4).

#### Health-related quality of life

The SF-12 (short-form health survey) has been found to be a valid and reliable measure of, and is widely used, to evaluate health status and HRQOL, including among older adults (Jakobsson, 2007). The SF-12 items assess several domains, and scoring on these 12 items can be combined to produce two summary scores:

- Physical Component Summary (PCS-12).
- Mental Component Summary (MCS-12).
  (Longitudinal data available for both.)

### Data Analysis

Where loneliness information was available, we conducted separate analyses for Māori (*n* = 254) and non-Māori (*n* = 398). Only participants with nonmissing data on variables of interest were included. Frequencies and percentages for categorical variables and means and standard deviations for normally distributed continuous variables were used to describe the two cohorts. We determined the cross-sectional prevalence of loneliness by sociodemographic characteristics at baseline. We then tested whether single sociodemographic or subjective well-being outcome items at baseline were associated with loneliness using the chi-square test for categorical variables and the *t* test for continuous variables (*p* < .05). Then, again at baseline, we conducted a series of multivariable models where each model, with a single outcome, included loneliness and all sociodemographic variables. For binary outcomes, odds ratio (OR), 95% confidence interval (CI), and *p* value are reported from logistic regression models. For normally distributed continuous outcomes, marginal mean, standard error, and *p* value are reported from multiway analysis of variance models.

For longitudinal person-year data, we first examined patterns of stability and change in loneliness. We then used mixed-effects models—with otherwise similar specifications to the baseline multivariable models—to account for repeated measures over time (up to six data waves for each person), that is, this adjusts for nesting of data from multiple waves within persons and gives an overall impact of loneliness on the outcomes. Here, the mixed effects refer to the fixed effects of loneliness and other predictors, and the random effect of the person. The mixed-effects technique is widely used to analyze longitudinal data and can utilize all available data while being robust to missing data (missing at random assumed) in any particular wave (Singer & Willett, 2003). SAS 9.4 software was used for data manipulation and analysis (SAS Institute Inc., 2016).

To assess the pattern of attrition (drop out or death): (a) we tracked loneliness prevalence across waves for those participants who completed Wave 6; (b) we tested if baseline loneliness prevalence was associated with loss to follow-up by Wave 6; and (c) we compared baseline sociodemographic characteristics of participants with and without Wave 6 data. We also examined the impact of attrition by excluding those without Wave 6 data and repeating the longitudinal analyses. These results are contained in Supplementary Tables S1, S2, and S4, and Supplementary Figure S1.

## Results

At baseline, the prevalence of loneliness (felt lonely ever) was 39.8% for Māori and 28.1% for non-Māori (Table 1). There were missing loneliness data for less than 3% of the combined sample in Wave 1.

**Table 1.** Baseline (2010) Loneliness by Sociodemographics and Outcome Measures, by Ethnic Group

	Māori					Non-Māori				
	All	Lonely	Not lonely			All	Lonely	Not lonely		
	*n* (%)^a^	*n* (%)^a^	*n* (%)^a^	All (% lonely)^b^	*p* Value	*n* (%)^a^	*n* (%)^a^	*n* (%)^a^	All (% lonely)^b^	*p* Value
Overall	254	(101) 39.8	(153) 60.2			398	112 (28.1)	286 (71.9)		
Sociodemographics										
Age group					.0236*					
80–85	206 (81.1%)	74.3	85.6	36.4		(all 85)	NA	NA	-NA	NA
≥85	48 (18.9%)	25.7	14.4	54.2						
Gender					.6435					.1874
Men	100 (39.4%)	37.6	40.5	38.0		188 (47.2%)	42.0	49.3	25.0	
Women	154 (60.6%)	62.4	59.5	40.9		210 (52.8%)	58.0	50.7	31.0	
Highest educational qualification					.4253					.9007
None/primary	72 (29.0%)	33.7	26.0	45.8		62 (15.9%)	16.8	15.5	29.0	
Secondary	139 (56.1%)	52.0	58.7	36.7		219 (56.0%)	54.2	56.7	26.5	
Postsecondary	37 (14.9%)	14.3	15.3	37.8		110 (28.1%)	29.0	27.8	28.2	
Main family occupation					.6838					.8516
Professional	106 (41.7%)	40.6	42.5	38.7		197 (49.5%)	48.2	50.0	27.4	
Technical/trade	45 (17.7%)	15.8	18.9	35.6		85 (21.4%)	23.2	20.6	30.6	
Other	103 (40.5%)	43.6	38.6	42.7		116 (29.2%)	28.6	29.4	27.6	
Marital status					.0129*					<.0001*
Partnered	83 (33.1%)	22.2	40.1	26.5		176 (44.7%)	22.7	53.2	14.2	
Widowed	151 (60.2%)	69.7	54.0	45.7		184 (46.7%	70.9	37.3	42.4	
Separated/divorced/never partnered	17 (6.8%)	8.1	5.9	47.1		34 (8.6%)	6.4	9.5	20.6	
Retired from paid work					.0082*					.6163
No	42 (17.1%)	9.3	22.3	21.4		90 (22.8%)	24.6	22.2	30.0	
Yes	203 (82.9%)	90.7	77.7	43.4		304 (77.2%)	75.5	77.8	27.3	
General well-being										
Life satisfaction					<.0001*					<.0001*
High (very satisfied)	66 (26.3%)	13.0	35.1	19.7		126 (32.3%)	13.0	39.7	11.1	
Not	185 (73.7%)	87.0	64.9	47.0		264 (67.7%)	87.0	60.3	35.6	
Quality of life					.0022*					<.0001*
High (very good)	86 (34.3%)	23.0	41.7	26.7		143 (36.5%)	16.7	44.0	12.6	
Not	165 (65.7%)	77.0	58.3	46.7		249 (63.5%)	83.3	56.0	36.1	
Health-related quality of life										
Physical (PCS-12)					.9979					.0875
*n*	244	97	147	NA		386	109	277	NA	
Mean (*SD*)	43.3 (11.1)	43.3 (10.3)	43.3 (11.7)			41.3 (12.1)	39.7 (11.0)	42.0 (12.4)		
Mental (MCS-12)					.0020*					<.0001*
*n*	244	97	147	NA		386	109	277	NA	
Mean (*SD*)	53.4 (8.7)	51.3 (9.4)	54.8 (7.9)			55.1 (8.3)	51.2 (9.6)	56.6 (7.2)		

*Notes*: MCS-12 = Mental Component Summary; NA = not applicable; PCS-12 = Physical Component Summary; *SD* = standard deviation.

^a^Distribution of loneliness—reported numbers are of those participants who also had sociodemographic and outcome data, respectively.

^b^Percentage of participants in each sociodemographic category who were lonely.

*p < .05—chi-square test used for percentages, and *t* test used for means.

### Baseline Bivariate Associations

At baseline, the proportion of Māori who were lonely was higher in those aged 85+ years (54.2% vs 36.4%, *p* = .0236), and in the retired (43.4% vs 21.4%, *p* = .0082; Table 1). For both Māori (26.5% vs 45.7%, *p* = .0129) and non-Māori (14.2% vs 42.4%, *p* < .0001), those partnered experienced less loneliness than those widowed. For both ethnic groups, there was no association between loneliness and gender, education level, or occupation.

Baseline loneliness was related to general well-being and HRQOL (Table 1). Firstly, for both ethnic groups, those who were lonely were less likely than those not lonely to have reported: (a) higher life satisfaction (Māori: 13.0% vs 35.1%, *p* < .0001; non-Māori: 13.0% vs 39.7%, *p* < .0001); and (b) higher general quality of life (Māori: 23.0% vs 41.7%, *p* = .0022; non-Māori: 16.7% vs 44.0%, *p* < .0001). Secondly, for both ethnic groups, those who were lonely were assessed to have lower mean mental HRQOL than those not lonely (Māori: 51.3 vs 54.8, *p* = .0020; non-Māori: 51.2 vs 56.6, *p* < .0001), whereas there was no association between loneliness and physical HRQOL.

### Baseline Multivariable Analyses

Table 2 shows the results of multivariable models where each model comprised loneliness as the predictor, adjusted for sociodemographic factors.

**Table 2.** Baseline (2010) Outcomes by Ethnic Group: Multivariable Models With Loneliness as Predictor of Interest

Outcome	Māori		Non-Māori	
	Odds ratio (95% CI)	*p* Value	Odds ratio (95% CI)	*p* Value
General well-being^a^^,^^c^				
Life satisfaction	(*n* = 242)		(*n* = 382)	
High (very satisfied/not)	0.26 (0.12–0.55)^d^	.0004*	0.21 (0.11–0.39)	<.0001*
	1.00 (reference: not lonely)		1.00 (reference: not lonely)	
Quality of life	(*n* = 242)		(*n* = 383)	
High (very good/not)	0.41 (0.22–0.77)	.0059*	0.27 (0.15–0.48)	<.0001*
	1.00 (reference: not lonely)		1.00 (reference: not lonely)	

	Marginal mean (*SE*)	*p* Value	Marginal mean (*SE*)	*p* Value
Health-related quality of life^b^^,^^c^				
Physical (PCS-12)	(*n* = 235)	.9344	(*n* = 379)	.1162
Lonely	45.2 (1.8)		39.3 (1.5)	
Not lonely	45.1 (1.5)		41.6 (1.1)	
Mental (MCS-12)	(*n* = 235)	.0002*	(*n* = 379)	<.0001*
Lonely	50.9 (1.4)		50.6 (1.0)	
Not lonely	55.4 (1.2)		56.2 (0.7)	

*Notes*: CI = confidence interval; MCS-12 = Mental Component Summary; PCS-12 = Physical Component Summary; *SE* = standard error.

^a^Logistic regression model of subjective well-being outcome variable with loneliness status as the predictor of interest (Wave 1, 2010): Māori aged 80–90, non-Māori aged 85.

^b^Multiway analysis of variance model of health-related quality of life outcome variable with loneliness status as the grouping of interest.

^c^All models are adjusted for sociodemographic variables (gender, education level, main family occupation, marital status, and retirement status—also age for Māori submodel).

^d^This can be interpreted as: Māori who were lonely had 0.26 times lower odds of having high life satisfaction compared with Māori who were not lonely.

*p < .05—Wald chi-square test used for odds ratios, and *F* test used for marginal means.

Firstly, for both Māori and non-Māori, the bivariate relationship found between loneliness and general well-being persisted. Being lonely, compared to not being lonely, conferred much lower odds of having (a) high life satisfaction (Māori: OR 0.26, 95% CI 0.12–0.55, *p* = .004; non-Māori: OR 0.21, 95% CI 0.11–0.39, *p* < .0001), and (b) high general quality of life (Māori: OR 0.41, 95% CI 0.22–0.77, *p* = .0059; non-Māori: OR 0.27, 95% CI 0.15–0.48, *p* < .0001).

Secondly, for both ethnic groups, the bivariate relationship between loneliness and HRQOL also persisted. The lonely group, compared to the not lonely group, was assessed to have lower mean mental HRQOL (Māori: 50.9 vs 55.4, *p* = .0002; non-Māori: 50.6 vs 56.2, *p* < .0001), while there was no association between loneliness and physical HRQOL.

### Longitudinal Trends

Investigation of attrition showed the following: (a) the pattern of loneliness prevalence across waves for those participants who completed Wave 6 was similar to that for all participants in each wave (without a clear trend; Supplementary Table S1 and Supplementary Figure S1); (b) for Māori, the baseline prevalence of loneliness in those who had Wave 6 data compared to those who did not was 33.3% versus 41.8%, respectively (chi-square = 1.3562, 1 df, *p* = .2442); for non-Māori, base prevalence on the same comparison was 28.7% versus 27.8% (chi-square = 0.0349, 1 df, *p* = .8518; Supplementary Table S2); and (c) Māori participants not progressing to Wave 6 were older, less educated, with technical occupations, and widowed; while for non-Māori, the sociodemographic profiles were broadly similar for those lost to follow-up and responders (Supplementary Table S2).

**Figure 1.**
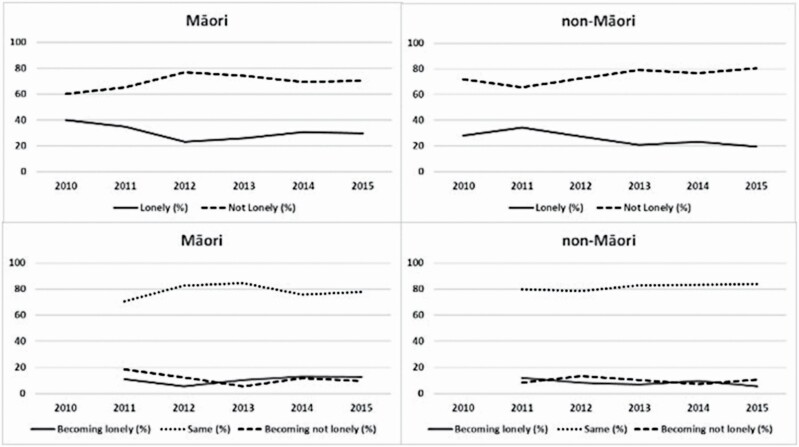
Proportion of those remaining in the study that were lonely and transition in loneliness status.

Figure 1 shows the degree of stability and change in loneliness across the six data waves. Flux between waves meant that of those reaching 5-year follow-up, 46.5% of Māori and 58.5% of non-Māori were in the same state as at outset.

For Māori, the prevalence of loneliness was 39.8% in 2010, lower in the next 2 years, followed by a rise until reducing to 29.6% in 2015, an overall decrease over 6 years (Figure 1; Supplementary Table S3). In terms of stability, we can see that, on average, 78.1% of Māori participants stayed in the same state as in the previous year, while 10.4% reported becoming lonely and 11.5% reported becoming not lonely.

For non-Māori, the prevalence of loneliness was 28.1% in 2010, with prevalence reducing to 19.5% in 2015 (Figure 1; Supplementary Table S3). On average, 81.6% of non-Māori participants remained in the same state as in the previous year, with 8.5% reporting becoming lonely and 10.0% reporting becoming not lonely.

### Longitudinal Multivariable Analyses

Table 3 shows the results of mixed-effects models. Each multivariable model, with loneliness as the predictor, was adjusted for data wave and sociodemographic factors.

**Table 3.** Longitudinal (2010–2015) Outcomes by Ethnic Group: Multivariable Mixed-Effects Models With Loneliness as Predictor of Interest

Outcome^a^	Māori		Non-Māori	
	Odds ratio (95% CI)	*p* Value	Odds ratio (95% CI)	*p* Value
General well-being^b^^,^^d^				
Life satisfaction	0.35 (0.21–0.59)	<.0001*	0.39 (0.26–0.58)	<.0001*
High (very satisfied/not)	1.00 (reference: not lonely)		1.00 (reference: not lonely)	

	Marginal mean (*SE*)	*p* Value	Marginal mean (*SE*)	*p* Value
Health-related quality of life^c^^,^^d^				
Physical (PCS-12)		.2743		.0196*
Lonely	42.4 (1.3)		38.4 (0.9)	
Not lonely	43.2 (1.1)		39.8 (0.8)	
Mental (MCS-12)		.0009*		<.0001*
Lonely	52.7 (1.0)		53.2 (0.6)	
Not lonely	55.0 (0.8)		56.0 (0.5)	

*Notes*: CI = confidence interval; MCS-12 = Mental Component Summary; PCS-12 = Physical Component Summary; *SE* = standard error.

^a^Using longitudinal data across a maximum of six waves per person; at baseline (Wave 1, 2010): Māori aged 80–90, non-Māori aged 85.

^b^Mixed-effects model of subjective well-being outcome variable with loneliness status as the predictor of interest.

^c^Mixed-effects model of health-related quality of life outcome variable with loneliness status as the grouping of interest.

^d^All models are adjusted for “wave” and sociodemographic variables (gender, education level, main family occupation, marital status, and retirement status—also age for Māori submodel); note that gender, education level, main family occupation, and retirement status were available at baseline only, while marital status was available longitudinally.

*p < .05—Wald chi-square test used for odds ratios, and *F* test used for marginal means.

Firstly, for both ethnic groups, the baseline relationship we found between loneliness and life satisfaction in cross-sectional multivariable models persisted in the longitudinal versions: being lonely, compared to not being lonely, conferred a lower likelihood of having high life satisfaction (Māori: OR 0.35, 95% CI 0.21–0.59, *p* < .0001; non-Māori: OR 0.39, 95% CI 0.26–0.58, *p* < .0001). Note that data for the general quality of life measure were available only at baseline.

Secondly, for both ethnic groups, the baseline relationship between loneliness and mental HRQOL also persisted in analogous longitudinal analyses: the lonely group, compared to the not lonely group, was assessed to have lower mean mental HRQOL (Māori: 52.7 vs 55.0, *p* = .0002; non-Māori: 53.2 vs 56.0, *p* < .0001). However, the longitudinal model found a significant relationship between loneliness and physical HRQOL in non-Māori but not in Māori: the lonely group, compared to the not lonely group, was assessed to have lower mean physical HRQOL (38.4 vs 39.8, *p* = .0196).

Sensitivity analyses that excluded those without Wave 6 data showed largely similar findings except that the association between loneliness and physical HRQOL for non-Māori was no longer statistically significant (Supplementary Table S4).

## Discussion

New Zealand is a demographically aging society with the subpopulation of older adults aged 80 years or older growing rapidly, both proportionally and in absolute numbers. We investigated the association between loneliness and subjective well-being in a unique bicultural cohort of adults in advanced age, with separate analyses for Māori and non-Māori. Mixed-effects models of well-being outcomes examined loneliness over time, adjusting for data wave and sociodemographic factors. Knowing the effects of loneliness on well-being will help inform the design of policy interventions to improve life outcomes for adults in advanced age.

### Principal Findings

#### Prevalence at baseline and over time

The baseline prevalence (2010) of loneliness in Māori and non-Māori groups was broadly similar to findings from the NZ General Social Survey (2012–2013), where 21.1% of adults aged 75 or older had felt lonely ever in the last 4 weeks (Statistics New Zealand, 2013), and to a 2016–2018 retirement village study of mostly non-Māori residents where 37.4% felt lonely ever (Boyd et al., 2020). In England, the “Newcastle 85+ Study” showed that, at baseline (2006), 43.3% of 85-year-olds felt lonely ever (Brittain et al., 2017).

We found no clear trend in loneliness with flux through the study for both Māori and non-Māori. Within this pattern of change, we can see stability in the proportion of participants who remained in the same state from year to year (78.1% for Māori, and 81.6% for non-Māori), though this proportion was reduced for those who remained either lonely or not lonely at 5-year follow-up (46.5% for Māori, and 58.5% for non-Māori). There are also indications that individuals can recover from loneliness: 11.5% of Māori participants and 10% of non-Māori participants reported becoming not lonely compared to the previous year. The Newcastle study found that, across a three-wave period of follow-up, 14.5% were “always” lonely, and 43.4% were “never” lonely, that is, 57.9% did not change their status over time (Brittain et al., 2017). Our study shows gross changes in the level of reported loneliness over time while simultaneously showing much stability in individual states. These findings underline loneliness as a dynamic process and the complexities involved in investigating well-being outcomes of loneliness.

#### Baseline bivariate associations

Marital status was significantly related to loneliness for both ethnic groups while retirement status was significant for Māori but not non-Māori; and there was no association between loneliness and gender, education level, or main family occupation. Here, we found similarity to previous studies of older adults, which showed that those with a partner are strongly protected from feeling lonely likely due to the intimate and supportive nature of such a relationship (e.g., Boyd et al., 2020; Cohen-Mansfield et al., 2016). Being retired from the workforce, particularly involuntarily, has also been associated with greater loneliness while being alleviated by social support (Shin et al., 2020). In this paper, sociodemographic factors were used to adjust the effect of loneliness on well-being outcomes in multivariable analyses.

At baseline, loneliness was negatively associated with life satisfaction and general quality of life, for both Māori and non-Māori. At baseline, loneliness was negatively associated with mental HRQOL but not with physical HRQOL, and these findings were consistent in both Māori and non-Māori.

#### Baseline multivariable analyses

The effects of loneliness found in baseline bivariate analyses persisted after adjusting for sociodemographic factors. Effect sizes were marked across life satisfaction and general quality of life, and moderate for mental HRQOL, and were similar for both ethnic groups. These findings build on research in younger old groups and support the notion that loneliness remains significant through advanced age. HRQOL was robust with mental HRQOL scores above 50 and physical HRQOL scores in the 40s. Adjusted physical HRQOL scores for Māori increased to around 45 suggesting resilience in physical health relative to greater socioeconomic burden.

#### Longitudinal multivariable analyses

There were longitudinal data for loneliness, marital status, life satisfaction, and mental and physical HRQOL. Findings from mixed-effects models of well-being outcomes (adjusted for sociodemographic factors) were mostly similar to baseline multivariable analyses with loneliness continuing to show strong effects for both ethnic groups. The exception was that loneliness became significantly related to physical HRQOL, though the association was slight and only detected for non-Māori (Māori had a smaller sample size). Sensitivity analyses, excluding those lost to follow-up, showed similar results, except that, for non-Māori, loneliness and physical HRQOL were no longer significantly associated.

### Implications

As far as we know, our study is the only one focusing on loneliness and well-being in a population-based group of this age, along with unique bicultural and longitudinal aspects. Given the paucity of evidence, our findings would be useful for policymaking and intervention. We found that loneliness was detrimental to general well-being and this is corroborated by cross-sectional studies of older adults broadly defined (e.g., Beller & Wagner, 2018). Cross-sectional studies have also found that loneliness impaired HRQOL in older adults (e.g., Tan et al., 2020). In a Finnish longitudinal study of older community-dwellers, Nummela et al. (2011) found that not only the absence of loneliness but also its decrease over time predicted good SRH. In longitudinal analyses, we found that loneliness affected mental HRQOL but not physical HRQOL of Māori in advanced age, whereas both HRQOL components were affected in non-Māori; we ascribe this finding to cultural differences, though the mechanisms are unclear and require further investigation. Another study of kaumātua (Māori older people) found that loneliness was negatively associated with both mental and physical HRQOL (Oetzel et al., 2019), indicating the importance of connection to whānau (family). A Swedish study of caregivers aged 75+ (EkwaIl et al., 2005) also found that loneliness was related to mental HRQOL but not physical HRQOL. Other studies of older adults give insights into how the link between loneliness and well-being might be broken, for example, by strengthening family ties (Holtfreter et al., 2016), by improving mental health and resilience (Gerino et al., 2017), or improving access to material resources (Szabo et al., 2019). There is also much evidence that higher levels of informal social activity—countering loneliness—are associated with better well-being in later life (Adams et al., 2011). Primarily, our finding that loneliness may affect the well-being of those in advanced age—so that preventing or ameliorating loneliness may improve well-being—argues for intervention on loneliness as a priority for public policy (Holt-Lunstad et al., 2017).

### Ethnic Differences

Although this paper is not focused on a direct comparison of the two ethnic groups in our study, we briefly address possible explanations for any differences (Dyall et al., 2014). The age distributions are different here, with Māori being younger, though, because Māori have a lower life expectancy, both groups were considered of “advanced age.” We found a higher level of loneliness in Māori than in non-Māori. Internationally, the finding that loneliness in later life is more prevalent among ethnic minority and immigrant groups than in the mainstream population has been attributed to ethnic differences in the effect of risk factors (Visser & El Fakiri, 2016). It has been argued that cultural meanings may shape perceptions and experiences of loneliness (van Staden & Coetzee, 2010). In the New Zealand case, Māori are the Indigenous people, though they form a minority of the national population, particularly in older age groups. Differences in loneliness between Māori and non-Māori may largely be due to: greater disadvantage that has arisen from the Māori experience of colonization and alienation (Reid et al., 2019); and unfulfilled expectations of social connection resulting from conflict between traditional, collective and modern, individualistic values (Brougham & Haar, 2013). Thus, it is crucially important, in modern ethnically diverse societies, such as bicultural New Zealand, to consider the cultural context when designing policies or interventions to prevent or alleviate loneliness (Morgan et al., 2020b), or to improve well-being (Oetzel et al., 2019). Loneliness had less impact on physical HRQOL for Māori than for non-Māori, adjusted for age and sociodemographic factors. Cultural customary concepts may provide resilience to disparities (Durie, 2011), and thus the way these associations play out could be expected to differ and suggests ethnic-specific responses.

### Strengths and Limitations

LiLACS NZ is the first study of its kind in New Zealand, creating a unique data resource for those in advanced age. The main strengths of this paper lie in the bicultural cohort and related longitudinal data covering a range of well-being indicators previously linked with loneliness. The sample size was relatively small and unable to detect an expected association between loneliness and education. Assessment of attrition showed no evidence that those lost to follow-up had been more lonely than those who remained in the study. However, disadvantaged subgroups were overrepresented among those lost to follow-up, which may indicate that the prevalence and effects of loneliness could be underestimated. We controlled for sociodemographic factors in the mixed-effects models, which may have mitigated this. Further, in simulation studies, Twisk et al. (2013) found that this type of model could handle missing data—including those due to attrition—to the extent that multiple imputation was not considered necessary.

Our measure of loneliness was based on a single direct item that is widely accepted and used (Victor et al., 2009). It elicits a subjective response to the question of being lonely, is easier to interpret, and enables quantifying the prevalence of loneliness. However, there are drawbacks: (a) it assumes a common understanding of what it is to be lonely, without an explicit definition or considering cultural differences, and (b) it may lead to bias (i.e., underreporting) due to social desirability or stigma surrounding the experience of loneliness. For purposes of analysis and reader interpretation, our measure was dichotomized (lonely or not) with loss of information. More in-depth understandings of the concept of loneliness may require qualitative methods with interpretation from a Māori worldview.

In our statistical models exploring the effect of loneliness on well-being, we assumed a unidirectional predictive relationship, and, while we adjusted for a range of sociodemographic factors, other possible confounders were not considered. We analyzed the longitudinal effect of loneliness on each well-being outcome, but—due to data limitations—were unable to model change in outcome as a function of change in loneliness.

### Future Research

The wider context of loneliness needs to be considered in order to inform effective interventions. Larger studies that facilitate subgroup analysis might better identify sections of society that are particularly vulnerable to loneliness and its harmful consequences for well-being. More sophisticated longitudinal modeling would also be useful for policy and practice, for example, analyzing transitions in loneliness in tandem with changes in outcomes over time. Consideration of the potential for strategies to prevent and assist recovery from loneliness may be informed by observational studies such as ours.

## Conclusion

This paper estimated the prevalence of loneliness and examined the baseline and longitudinal relationships between loneliness and subjective well-being outcomes in Māori and non-Māori in advanced age from one region in New Zealand in the 2010s. Most effects of loneliness persisted and were stable over time. Our findings generally accord with the literature and thus further highlight loneliness for people in advanced age as a prime candidate for intervention to improve their well-being.

## Supplementary Material

gbac087_suppl_Supplementary_Material
