# Supplementary material for: Prevalence of Loneliness and Its Association With General and Health-Related Measures of Subjective Well-Being in a Longitudinal Bicultural Cohort of Older Adults in Advanced Age Living in New Zealand: LiLACS NZ
Source: J Gerontol B Psychol Sci Soc Sci. 2022 Jun 29;77(10):1904–15. doi: 10.1093/geronb/gbac087 (PMC9535776; doi:10.1093/geronb/gbac087)
Supplement: gbac087_suppl_Supplementary_Material [file gbac087_suppl_supplementary_material.pdf]

Prevalence of loneliness and its association with general and health-related measures of subjective well-being in a longitudinal bicultural cohort of older adults in advanced age living in New Zealand: LiLACS NZ

Supplementary Material

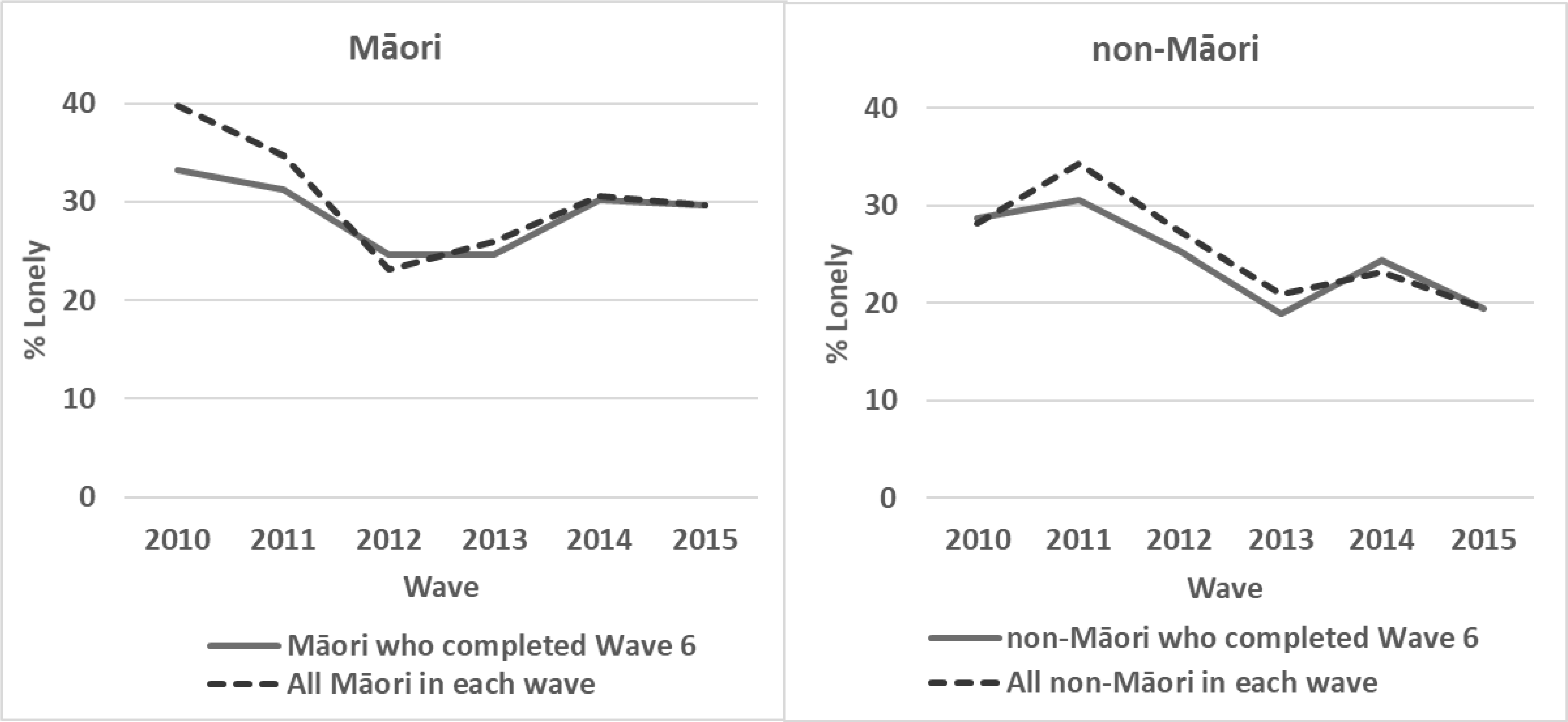

Figure S1. Prevalence of loneliness across Waves 1-6: for participants who completed Wave 6 versus all participants in each wave, by ethnic group

Table S1. Prevalence of loneliness across Waves 1-6: for participants who completed Wave 6 versus all participants in each wave, by ethnic group

	Māori						Non- Māori					
	2010	2011	2012	2013	2014	2015	2010	2011	2012	2013	2014	2015
	**Completed Wave 6 (n=71)**						**Completed Wave 6 (n=164)**					
Lonely (%)	33.3	31.3	24.6	24.6	30.2	29.6	28.7	30.6	25.3	18.9	24.4	19.5
	**All participants in each wave**						**All participants in each wave**					
Lonely (%)	39.8	34.7	23.1	25.9	30.6	29.6	28.1	34.3	27.4	20.9	23.2	19.5
n (denominator)	254	222	156	116	85	71	398	365	303	244	198	164

Table S2. Wave 1 (2010) loneliness prevalence and socio-demographic characteristics of participants who did and did not have loneliness data in Wave 6 (2015), by ethnic group

	Wave 6 loneliness data			
	Available (n=235) [a]		Missing (n=435)	
	**Māori** n=71	**Non-Māori** n=164	**Māori** n=194	**Non-Māori** n=241
	n (%) [b]	n (%)	n (%)	n (%)
**Wave 1 -Loneliness prevalence**	20 (33.3)	45 (28.7)	81 (41.8) [c]	67 (27.8) [c]
			X^2=1.3562, 1 df, p=0.2442	X^2=0.0349, 1 df, p=0.8518
**Wave 1 - socio-demographics**				
Age group		(all 85)		(all 85)
80-85	59 (83.1%)		152 (78.4%)	
>=85	12 (16.9%)		42 (21.6%)	
Gender				
Men	27 (38.0%)	78 (47.6%)	78 (40.2%)	113 (46.9%)
Women	44 (62.0%)	86 (52.4%)	116 (59.8%)	128 (53.1%)
Highest educational qualification				
None/primary	13 (18.8%)	26 (15.9%)	61 (32.3%)	39 (16.6%)
Secondary	51 (73.9%)	94 (57.7%)	96 (50.8%)	128 (54.5%)
Post-secondary	5 (7.3%)	43 (26.4%)	32 (16.9%)	68 (28.9%)
Main family occupation				
Professional	28 (39.4%)	85 (51.8%)	78 (40.2%)	112 (46.5%)
Technical/trade	9 (12.7%)	31 (18.9%)	36 (18.6%)	54 (22.4%)
Other	34 (47.9%)	48 (29.3%)	80 (41.2%)	75 (31.1%)
Marital status				
Partnered	30 (42.2%)	78 (47.8%)	56 (29.3%)	101 (42.4%)
Widowed	34 (47.9%)	73 (44.8%)	124 (64.9%)	114 (47.9%)
Separated/divorced/never partnered	7 (9.9%)	12 (7.4%)	11 (5.8%)	23 (9.7%)
Retired from paid work				
No	11 (18.6%)	32 (20.5%)	31 (16.7%)	58 (24.4%)
Yes	48 (81.4%)	124 (79.5%)	155 (83.3%)	180 (75.6%)

a. 19 participants did not have Wave 1 loneliness data; b. reported numbers are of those participants who had loneliness, socio-demographic and outcome data respectively; c. chi-square test of the association between baseline loneliness and loss to follow up by Wave 6, i.e., is there a difference in baseline loneliness between those with and without Wave 6 data

Table S3. Stability and change in loneliness across Waves 1-6 (2010-2015), by ethnic group

	Māori						Non-Māori					
	2010	2011	2012	2013	2014	2015	2010	2011	2012	2013	2014	2015
**Prevalence**												
n (denominator)	254	222	156	116	85	71	398	365	303	244	198	164
Lonely (%)	39.8	34.7	23.1	25.9	30.6	29.6	28.1	34.3	27.4	20.9	23.2	19.5
Not Lonely (%)	60.2	65.3	76.9	74.1	69.4	70.4	71.9	65.7	72.6	79.1	76.8	80.5
**Stability and change** [a]												
Becoming lonely (%)	-	11.0	5.4	10.2	12.8	12.7	-	11.9	8.2	6.9	9.7	5.6
Same (%) [b]	-	70.5	82.3	84.3	75.6	77.8	-	79.8	78.4	82.8	83.1	83.8
Becoming not lonely (%)	-	18.5	12.2	5.6	11.5	9.5	-	8.3	13.4	10.3	7.2	10.6
		100%	100%	100%	100%	100%		100%	100%	100%	100%	100%
Remained in baseline state (lonely or not)	46.5%						58.5%					

a. change since previous wave; b. same = remained in previous state (whether lonely or not)

Table S4. Longitudinal (2010-2015) outcomes for participants who had loneliness data in Wave 6, by ethnic group: Multivariable mixed effects models with loneliness as predictor of interest

Outcome [a]	**Māori (n=59)**		**Non-Māori (n=155)**	
	Odds Ratio (95% CI)	p	Odds Ratio (95% CI)	p
**General well-being** [bd]				
Life satisfaction	0.31 (0.12-0.82)	0.0192 *	0.49 (0.27-0.87)	0.0144 *
High (very satisfied/not)	1.00 *(reference: not lonely)*		1.00 *(reference: not lonely)*	
	Marginal mean (se)	p	Marginal mean (se)	p
**Health-related quality of life** [cd]				
Physical (PCS-12)		0.9367		0.7591
Lonely	44.2 (2.2)		42.0 (1.2)	
Not lonely	44.2 (2.0)		42.2 (1.1)	
Mental (MCS-12)		0.0259 *		0.0003 *
Lonely	52.7 (1.6)		54.2 (0.8)	
Not lonely	54.6 (1.4)		56.4 (0.6)	

a. using longitudinal data across up to 6 waves per person; b. mixed effects model of subjective well-being outcome variable with loneliness status as the predictor of interest; c. mixed effects model of HRQOL outcome variable with loneliness status as the grouping of interest; d. all models are adjusted for ‘wave’ and socio-demographic variables (gender, education level, main family occupation, marital status, and retirement status - also age for Māori sub-model); note that gender, education level, main family occupation, and retirement status were available at baseline only, while marital status was available longitudinally;
* $p<.05$ - Wald chi-square test used for odds ratios, and F test used for marginal means
